# A Cross-International Study to Evaluate Knowledge and Attitudes Related to Basic Life Support among Undergraduate Nursing Students—A Questionnaire Study

**DOI:** 10.3390/ijerph17114116

**Published:** 2020-06-09

**Authors:** Katarzyna Kwiecień-Jaguś, Wioletta Mędrzycka-Dąbrowska, Nijolė Galdikienė, Gemma Via Clavero, Monika Kopeć

**Affiliations:** 1 Department of Anaesthesiology and Intensive Care Nursing, Medical University of Gdańsk, 80-210 Gdańsk, Poland; wioletta.medrzycka-dabrowska@gumed.edu.pl; 2 Department of Strategig Development, Klaipėda State University of Applied Sciences, 91274 Klaipėda, Lithuania; n.galdikiene@kvk.lt; 3 Department of Fundamental Care and Medical-Surgical Nursing, University of Barcelona, 08007 Barcelona, Spain; gviac@ub.edu; 4 Department of Human Nutrition, University of Warmia and Mazury, 10-718 Olsztyn, Poland; monika.kopec@uwm.edu.pl

**Keywords:** cardiopulmonary resuscitation (CPR), nurse education, nursing students, European Resuscitation Council (ERC)

## Abstract

Practicing the algorithms of basic life support (BLS) is essential in order to take adequate measures to save lives, and future nursing staff should be advanced when it comes to performing lifesaving activities. The purpose of the study was to analyse the theoretical and practical knowledge of nursing students (within the undergraduate course) with regards to the basic life support (BLS). The study has a prospective, comparative and descriptive nature. Three countries that educate nursing students within the undergraduate course participated in the project. Data was collected with the use of a questionnaire based on the guidelines of the European Resuscitation Council (ERC 2015). The study was carried out among a total of 748 respondents from three countries: Poland (*n* = 189, 25.3%), Lithuania (*n* = 500, 66.8%) and Spain (*n* = 59; 7.9%). The average age of respondents was 23.41 (SD ± 5.90). The average score obtained by the respondents who answered the questions was 11.13 with a standard deviation of SD ± 2.29. The conducted study revealed that a substantial number of the respondents who participated in the study obtained an average result concerning theoretical and practical knowledge of BLS. It was also confirmed that the additional education in the form of first aid training courses has a major impact on improving the levels of knowledge among the students in each of the analysed country.

## 1. Introduction

The European Resuscitation Council is an organization involved in developing the most effective and practical rules of providing first aid as well as the advanced resuscitation procedures [1]. Cardio-pulmonary resuscitation (CPR) is one of the basic skills from the Basic Life Support (BLS) algorithm. By the definition, it is a collection of interventions performed to provide a proper amount of oxygen and circulation to the victim’s body during cardiac arrest. [2]. The information included in the guidelines is so universal that it can be applied not only in every European country but also in each centre that is involved in the education of a medical personnel at the university, within the research degree or during additional trainings. Available scientific studies revealed that nursing students as well as newly employed graduates of nursing do not always have the adequate skills or knowledge to provide proper resuscitation [3]. The Bologna Process divides the nursing programs education into three levels: bachelor, master and doctorate in nursing. A full academic offer is provided by 60% of the countries. Nevertheless, there are still countries leading education of nursing personnel only within the diploma program [4]. The situation is similar when it comes to achieving the proper knowledge and professional skills. Although the framework of educating nursing personnel in European countries is defined by the directives of the European Union [5,6] as well as by the WHO recommendations [7], there is still lack of common position. What is more, the process of how the nursing personnel is educated and the number of hours allocated to the subjects related to the BLS skill and knowledge are a very important issue in each European country.

In Poland, the education of the nursing personnel within the undergraduate course takes a minimum of 4720 h, including 2300 h of practical classes. Every university independently allocates the number of hours dedicated to specific subjects. BLS education is offered within the anaesthesiology and nursing in life-threatening conditions as well as emergency medicine. The National Accreditation Council requires at least 5% of specialist trainings in simulated environment. The simulation classes are mostly divided into three different areas: partial task, computer simulation and high-fidelity simulator in the Simulation Centre. During the partial task, students use different devices to learn highly specialised skills such as assisting with intubation, securing intubation tube, changing dressing on the central catheter, chest compression during the CPR and the management of the airway and ventilation. The computer simulation is an interactive program that allows the learners to practice patient care and get certain feedback on their medical management. The high-fidelity mannequin simulator (HFMS) is a dynamic, computer-controlled, full-sized, simulated mannequin capable of giving a history, recreating physical exam findings such as normal and abnormal heart sounds, lung sounds and pupil findings, as well as physiologic changes including blood pressure, heart rate and breathing [8,9,10,11]. 

In Spain, the number of theoretical and practical classes during the undergraduate course has to be at the level of at least 6100 h, including 2100 h of practical education. At least 3 h are dedicated to discussing issues related to the CPR algorithm within the subject of pathophysiology, including 1.5 h in simulated environment using manikins [12].

In Lithuania, the education of nursing personnel requires at least 4600 h, including 2300 h of practical classes. The allocation of hours as well as the achievement of educational effects depends on the internal rules of the centres involved in the training of the nursing personnel [4].

The aim of the study was to analyse the theoretical and practical knowledge of basic life support (BLS) among the undergraduate students. 

## 2. Materials and Methods

The study has a prospective, comparative and descriptive character. A total of 748 students of the undergraduate nursing studies from Lithuania, Poland and Spain participated in the study. The project was approved by the Bioethical Commission for Scientific Studies at the Medical University of Gdańsk (approval No NKBBN/36/2018) and the authorities of the universities that took part in the study. The participants of the study were informed about the aim and the assumptions of the project. The questionnaire was distributed via the Internet, and the participants did not have to log on or provide any personal data in relation to the project. The respondents gave their written, on-line consent to participate in the project. The researchers prepared an on-line version of a consent form which stated: “I agree to participate in the research study and fill the on-line questionnaire”. The questionnaire was prepared according to the guidelines of the European Resuscitation Council [13] and was divided into three parts. The first part included socio-demographic questions. The second part contained 10 questions that concerned the BLS and AED. Students were asked about the correct hand position place when performing chest compressions for adults and children, what would they do if somebody would show the symptoms of chocking, and what does the AED abbreviation mean. The third part of the survey consisted of 7 questions about the skills of performing lifesaving activities. Respondents were asked about the sequence of CPR algorithm, the ratio of chest compressions during CPR, the correct placement of AED pads and the sequence of the use of AED device. The students had to achieve at least 60–69% of the correct answers in order to pass the test at a sufficient level. A result between 70–79% meant that the students achieved a fairly good level of knowledge. A score above 80% was classified as a good level while one over 90% of correctness was estimated as a very good level of knowledge and skills. All the questions in the questionnaire were closed. One point was granted per correct answer. The score of 17 points indicated that all the answers were correct. Before the research team started the project, a pilot survey (in Polish) had been provided to a group of 10 nursing students and 5 professionals from the areas of critical care and emergency medicine. 

The questions were consulted with the students. Some of the questions and answers were modified according to the suggestions in order to give them more clarity. The experts approved the final version. The Polish version of the questionnaire was then translated into English and then back translated by two independent translators. The English version of the tool was then provided to two research team members located in Spain and Lithuania. It took one year to collect the research material.

### Statistical Methods

All statistical analyses were carried out using the IBM SPSS 23 statistical package (IBM SPSS^®^, Chicago, IL, USA) as well as an Excel 2016 spreadsheet (Microsoft Corporation, Redmond, WA, USA).

Quality variables were presented with the numbers and percentage values, while quantity variables were characterised with an arithmetic average and a standard deviation. The Kolmogorov–Smirnov (K-S) test was used to check if there were the normally distributed quantity variables. The significance of differences between two groups was checked by using the non-parametric U Mann–Whitney test. The differences between more than two groups were presented with the use of Kruskal–Wallis test. The post hoc test was used if major differences were obtained; in this case, it was Bonferroni test. The Spearman’s correlation test was applied to measure the strength and direction. However, a chi-squared test was implemented to examine the quality variables. Results were significant if *p* < 0.05.

## 3. Results

**The study included a total number of 748 respondents from three countries: Poland (*n* = 189, 25.3%**, Gdańsk University of Medicine), Lithuania (*n* = 500; 66.8%, Kauno Kolegija, Klaipedos Valstybine Kolegija, Panevezio Kolegija, Siauliu Valstybine Kolegija Utenos Kolegija, Vilniaus Kolegija) and Spain (*n* = 59; 7.9%, University of Barcelona). Respondents who participated in the study attended the following years of studies: 1st (*n* = 273; 36.5%), 2nd (*n* = 1; 0.2%), 3rd (*n* = 70; 9.4%) and 4th (*n* = 404; 54%). The analysed group was internally diversified in terms of gender, with a large group of female respondents (*n* = 709; 94.8%). The average age of the respondents was 23.41 (SD ± 5.90). More than 11.8% (*n* = 88) of the respondents declared that they had completed other healthcare studies before they started to study nursing. Every respondent declared attending a first aid training course, including 51% (*n* = 563) who had attended first aid training during a driving license course, a qualified first aid course (*n* = 61; 5.5%), secondary school classes (*n* = 277; 25.1%) as well as an obligatory training before starting a new work (*n* = 100; 9.1%). A vast majority of respondents had never provided a pre-medical aid, while 23.3% (*n* = 174) of the respondents declared they had provided aid in out-of-hospital situations. (Table 1).

**Figure 1:**
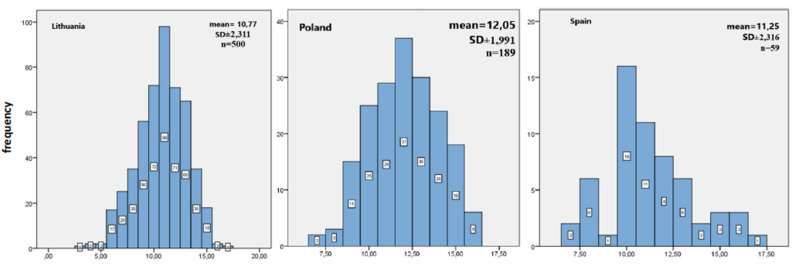
The distribution of the score points within the level of knowledge about CPR—Lithuania, Poland and Spain.

In order to determine respondents’ level of knowledge, the points from all the questions were summed up. Next, descriptive statistics was implemented to calculate the new variables. The results that could be obtained in the questionnaire measuring the level of knowledge and practical skills were in a range from 0 to 17. The lowest result obtained by respondents was *Min* = 3, and the highest was *Max* = 17. The average result of respondents was 11.13, with a standard deviation of *SD* ± 2.29. The classification of the results was not in line with the normal distribution, and it was confirmed by testing the normality of distribution K-S (*K-S*_(748)_ = 0.10; *p* < 0.05). Detailed analyses revealed that only two respondents in the group achieved 100% of correct responses. The 18.45% of respondents (*n* = 138) scored 11 points (Figure 1). A detailed analysis of correct answers given by the respondents showed that more than 66% of them (*n* = 496; 66.5%) knew the meaning of the BLS acronym, while 76.8% (*n* = 573) remembered that in the process of providing assistance the most important thing is to ensure the safety of the rescuer and the injured person. 76,4% (*n* = 570) of the respondents knew the right place to compress the chest of an adult and a child (*n* = 436; 58.8%). The answers regarding the correct depth of chest compressions were also at the satisfactory level (*n* = 523; 70.3%) as well as the frequency of the chest compressions for both adults and paediatric patients (*n* = 319; 42.9%). In another part concerning the knowledge of practical skills related to first aid, only 22.3% (*n* = 166) of the respondents knew the correct algorithm of basic resuscitation activities for an adult. More than 82.6% (*n* = 641) of the respondents knew how to assess the pulse of an adult, and 80.9% (*n* = 603) knew the correct ratio of chest compressions and rescue breaths. The question concerning the correct area of AED defibrillator electrodes on the chest of an injured person was not difficult (*n* = 437; 59.4%). What is more, the question about the frequency of assessing the condition of the injured person in the course of basic resuscitation measures (*n* = 496; 66.6%) also did not cause bigger issues. More than half of the respondents knew how to ensure their own safety as well as the safety of the injured person while using AED (*n* = 543; 76.6%). (Table 2)

In order to verify the difference between the groups, the analysis conducted with the U Mann–Whitney test showed that the respondents who had completed a first aid course while attending secondary school represented (statistically) significantly higher level of knowledge (Z = −3.85; *p* < 0.001). The analysis with the use of Spearman correlation test confirmed that higher BLS knowledge of the respondents was related to their advancement of education rHO = 0.23; *p* < 0.001 and the semester they were in rHO = 0.23; *p* < 0.001. (Table 3) The study did not show a significant relationship between the gender and the level of knowledge among nursing students (Z = −1.52; *p* >0.05). By using the Kruskal–Wallis non-parametric significance test, statistically significant dependencies were obtained between the variables H_(2)_ = 38.97; *p* > 0.001. The method of Bonferroni’s test was used to check if there is a significant dependence between any of the groups. Detailed analyses confirmed a significantly higher level of knowledge regarding BLS among students from Poland than from Lithuania. (Table 4) No statistically significant differences *p* > 0.05 were achieved between any other groups.

## 4. Discussion

The conducted study showed that the knowledge of students within the algorithms and guidelines of the European Resuscitation Council related to such knowledge as practical skills and BLS algorithms is at a good, although insufficient level. A significant number of students obtained only 11 out of 17 points, achieving 64.7% of the correct answers. Only two people from the group obtained the maximum score. The findings of the study are similar to the findings achieved by the other authors. Oteir led research among Jordanian students from different medical fields such as medical laboratory science, physical therapy, occupational therapy, speech pathology, dental technology, allied dental science, radiological technology and optometry. His research team used a very similar questionnaire based on the AHA guidelines from 2015. His results were based on a group of 1525 respondents and were very similar to our own studies. He proved that the knowledge and CPR skills are at a poor level among Jordanian students. The high expertise and abilities were mainly associated with previous CPR training or recent training [14]. The analysis of the data shows that the lack of opportunities related to consolidating basic rescue skills and knowledge impacts the level of BLS expertise of nursing students. Their level of knowledge is often below the minimum standards [3,15]. The authors of many, great publications point out that the main problem is the excessive number of classes and the fact that students are generally overloaded. Another quite important factor is the insufficient number of hours of simulation classes, which naturally extend the scope of soft skills and practical skills. The study of the rules of providing first aid, which students will be able to apply both in hospital conditions and in everyday life, has to include a practical training with classes in simulated environment on manikins [15,16]. Kanstad’s [16] analyses showed that the level of knowledge among the respondents increased with their age as well as due to previous first aid training, either at a secondary school or during other postgraduate forms of education/courses. The findings of this study correspond with the observations of other scientists indicating that BLS training should begin as soon as possible. What is more, it should be revised annually because this seems to be the most effective way of refreshing the skills [17].

The recommendations of the European Resuscitation Council are universal for every country, and the European Parliament has determined the framework of educating nursing personnel. Despite this, this project has indicated that there are significant differences between the countries and among the students with regards to their level of skills and BLS algorithm knowledge. The above-mentioned differences are certainly related to the internal standards of education and the curriculum of the medical studies. The lack of consistent requirements allows each country to decide when and how the ERC guidelines will be discussed during the studies. Countries where the education of basic rescue activities is continued and revised every year show a significantly higher level of knowledge when compared with the countries where the students learn about CPR issues within a single subject only. The lack of international research with regards to this topic and including such a study group does not allow to compare the findings with those of other researchers. R. Hamilton suggested in one of his studies that annual basic rescue activities training should be obligatory including the nurses with long work experience, especially in the departments which deal with few incidents of cardiac arrest [18].

## 5. Conclusions

The analysis of the collected data has allowed to formulate the following conclusions: The level of the BLS knowledge among the students remains at a medium level; the level of BLS knowledge is increasing together with the age of students and the semesters they completed. A significantly higher level of theoretical and practical knowledge was demonstrated by students who had attended additional training in BLS during driving license courses or at secondary schools. The level of knowledge among nursing students from Poland was significantly higher than among Lithuanian students.

The high level of knowledge as well as nursing students’ BLS skills are with no doubt connected to the frequency of training. In the future, it will be worth considering common European standards related to the education of CPR. Such standards should take into account not only the number of hours required to obtain the knowledge and expertise but also the equipment which would allow to run the classes in the simulation-based learning environment. Currently, there is a disparity in the number of hours dedicated to learning lifesaving skills across each country. The process of learning should be supported with the development of soft skills, including the cooperation and proper communication between the members of a medical team. Because of that, it is worth considering teaching BLS skills in diversified groups, including various professions and the level of advancement.

### Limitations of the Study

The project was limited by the selection of the group and with regards to the differences between individual countries in terms of the curriculum and hours within the undergraduate course. The difficulties that occurred during the project were related to the fact that there is no standardized survey regarding CPR knowledge; however, we based our study on the ERC 2015 recommendations as well as the proper literature findings. Despite the fact that the topic and skills are essential especially in terms of everyday hospital work, the studies that aim to evaluate medical students’ (including nursing) BLS knowledge and skills are still not at a sufficient level.

## Figures and Tables

**Table 1:** Socio-demographic characteristic of the nursing students.

Parameters	*n*	%
Country		
Poland	189	25.3
Lithuania	500	66.8
Spain	59	7.9
Gender		
Female	709	94.8
Male	39	5.2
Name of the school/university in which you study nursing		
Medical University of Gdańsk	189	25.3
Kauno Kolegija	91	12.2
Klaipedos Valstybine Kolegija	134	17.9
Panevezio Kolegija	28	3.7
Siauliu Valstybine Kolegija	82	11.0
University of Barcelona	59	7.9
Utenos Kolegija	49	6.6
Vilniaus Kolegija	116	15.5
Did you have any professional title in the area of healthcare before starting your nursing studies?		
Yes	88	11.8
No	660	88.20
Year of study		
First	273	36.5
Second	1	0.1
Third	70	9.4
Fourth	404	54.0
Semester of study		
1	65	8.7
2	262	35.0
4	1	0.1
6	70	9.4
7	346	46.3
8	4	0.5
Have you ever administered first aid in a non-hospital setting?		
Yes	174	23.3
No	574	76.7
Which first aid courses have you completed?		
First aid course being part of my driving course	563	51.0
Advanced first aid course	61	5.5
First aid course that I took as part of my secondary school curriculum	277	25.1
First aid course arranged by my employer	100	9.1
Other	103	9.3

**Table 2:** Knowledge and practice about basic life support among nursing student population.

Knowledge	*n*	%
What does the abbreviation BLS stand for?		
Best Life Support	141	18.9
Basic Life Support	496	66.5
Basic Lung Support	41	5.5
Basic Life Service	68	9.1
You find your friend in the middle of the road—he is unconscious, does not react, and does not breathe properly. What do you do first?		
I clear his airways	75	10.1
I make sure we are both safe	573	76.8
Begin to give him chest compressions	79	10.6
I give him two rescue breaths	19	2.5
Having confirmed that the victim does not react even to strong stimuli such as shaking or loud shouts for help, what will you do next?		
Call an ambulance	571	76.5
Perform cardiovascular resuscitation	107	14.3
Place him in the recovery position	25	3.4
Continue observations	43	5.8
The correct place for the compression of the chest in an adult is:		
The left side of the chest	30	4.0
The right side of the chest	9	1.2
The centre of the chest	570	76.4
The xiphoid process	137	18.4
The correct place for the compression of the chest in an infant is:		
Two fingers placed on the bottom part of the sternum	156	21.1
Two fingers placed in the area of the xiphoid process	93	12.6
Two fingers placed in the central part of the sternum on the breast line	436	58.8
Two fingers placed on the top part of the sternum	56	7.6
The correct depth for chest compression in children during BLS is:		
1/3 of the chest depth	523	70.3
1 and a half of the chest depth	51	6.9
1/2 of the chest depth	157	21.1
2 and a half of the chest depth	13	1.7
The correct frequency of chest compressions during CPR for adults and children is:		
At least 100 compressions per minute	317	42.9
More than 100 compressions per minute	85	11.5
80 compressions per minute	159	21.5
120 compressions per minute	178	24.1
What does the abbreviation AED stand?		
Automated External Defibrillator	231	31.0
Automated Electrical Defibrillator	410	55.1
Advanced Electrical Defibrillator	66	8.9
Advanced External Defibrillator	37	5.0
If your friend suddenly shows symptoms of choking, what will you do?		
Immediately hit his chest several times	133	17.9
Compress his epigastrium several times	419	56.2
Confirm choking by way of an interview	174	23.4
Give him two rescue breaths	19	2.6
Rescue breaths in an infant are performed:		
Mouth-to-mouth with the nostrils pinched closed	241	32.9
Mouth-to-mouth or mouth-to-nose	251	34.3
Only mouth-to-nose	105	14.3
Mouth-to-mouth without the nostrils pinched closed	135	18.4
**Practice**		
Which of the following is the algorithm for basic resuscitation in an adult?		
Make sure both you and the victim are safe, give two rescue breaths, perform defibrillation, begin CPR	40	5.4
Make sure both you and the victim are safe, call an ambulance, check for a pulse, begin CPR	509	68.5
Check for a pulse, give two rescue breaths, make sure both you and the victim are safe, perform defibrillation	28	3.8
Make sure both you and the victim are safe, begin CPR, give two rescue breaths, perform defibrilation	166	22.3
We check for a pulse in an adult on the:		
Carotid artery	641	86.2
Brachial artery	80	10.8
Femoral artery	10	1.3
Temporal artery	13	1.7
The ratio of chest compressions to breaths during CPR in adults is:		
15:2	73	9.8
15:1	37	5.0
30:1	32	4.3
30:2	603	80.9
The 2015 ERC guidelines concerning basic resuscitation operations recommend the following algorithm of procedure:		
Compress the chest, clear the airway, check the breathing	168	22.6
Clear the airway, check the breathing, check the pulse	50	6.7
Clear the airway, check the breathing, check the pulse	527	70.7
The correct placement of defibrillation electrodes from the AED set is on the front chest, always with the long axis parallel to the long axis of the body:		
One on the right side of the sternum, below the clavicle; the second one on the 5th intercostal space of the midaxillary line	437	59.4
The electrodes may be applied on the chest in any place on both sides of the victim’s sternum	67	9.1
One under the left clavicle along the sternum, the second one above the victim’s cardiac apex	193	26.2
The electrodes should be placed 2 fingers above the victim’s intercostal angle	39	5.3
During the basic resuscitation procedure, the condition of the victim should be assessed:		
Every minute	158	21.2
Every 5 cycles (30 chest compressions and 2 rescue breaths)	496	66.6
When the victim starts to breathe correctly	50	6.7
Before the attachment of the AED electrodes	41	5.5
Which of the following is the correct sequence of the use of AED defibrillator?		
Switch on the AED, apply the electrodes, discharge, analyse the rhythm	50	6.8
Switch on the AED, apply the electrodes, analyse the rhythm, make sure no one touches the victim, discharge	543	73.6
Apply the electrodes, check the pulse, discharge, analyse the rhythm	48	6.5
Check the pulse, apply the electrodes, analyse the rhythm, discharge	97	13.1

**Table 3:** Correlation between variables—The level of knowledge about basic life support (BLS) vs first aid course.

Parameter	n	M	SD	Z	*p*-Value
Level of knowledge about BLS vs first aid course being part of my driving course					
**yes**	185	11.09	2.49	0.22	0.82
**no**	563	11.14	2.23		
Level of knowledge about BLS vs advanced first aid course					
**yes**	686	11.12	2.28	0.47	0.64
**no**	61	11.22	2.44		
Level of knowledge about BLS vs. first aid course which during secondary school					
**yes**	471	10.89	2.35	3.85	0.000 *
**no**	277	11.53	2.14		
Level of knowledge about BLS vs first aid course arranged by employer					
**no**	648	11.13	2.31	0.05	0.955
**yes**	100	11.1	2.21		

Legend: Min, minimal; Max, maximal; M, mean; SD, standard deviation. U–Mann–Whitney test to determine the significant variables. * Significance of differences between the study groups, *p* < 0005.

**Table 4:** The multiple comparison between three countries—the level of knowledge about BLS.

The Level of Knowledge vs Country	*n*	M	SD	H	*df*	*p*-Value
Poland	189	12.05	1.99	38.97	2	0
Lithuania	500	10.77	2.31			
Spain/Barcelona	59	11.25	2.31			

Legend: Min, minimal; Max, maximal; M, mean; SD, standard deviation; df, degree of freedom, *p*-value; Bonferroni test, *p*-value > 0.05.

## Abbreviations

AED	Automated External Defibrillation
AHA	American Heart Association
BLS	Basic Life Support
CPR	Cardiopulmonary Resuscitation
ERC	European Resuscitation Council
HFMS	High-fidelity Mannequin Simulator
K-S	the Kolmogorov–Smirnov

## References

[1] Finn J.C., Bhanji F., Lockey A., Monsieurs K., Frengley R., Iwami T., Lang E., Ma M.H., Mancini M.E., McNeil M.A. (2015). Education, Implementation, Teams Chapter Collaborators. Part 8: Education, implementation, and teams: 2015 International Consensus on Cardiopulmonary Resuscitation and Emergency Cardiovascular Care Science with Treatment Recommendations. Resuscitation.

[2] Truong H.T., Low L.S., Kern K.B. (2015). Current Approaches to Cardiopulmonary Resuscitation. Curr. Probl. Cardiol..

[3] Méndez-Martínez C., Martínez-Isasi S., García-Suárez M., Peña-Rodríguez M.A., Gómez-Salgado J., Fernández-García D. (2019). Acquisition of Knowledge and Practical Skills after a Brief Course of BLS-AED in First-Year Students in Nursing and Physiotherapy at a Spanish University. Int. J. Environ. Res. Public Health.

[4] Lahtinen P., Leino-Kilpi H., Salminen S. (2014). Nursing education in the European higher education area—Variations in implementation. Nurse Educ. Today.

[5] EU Parliament and Council Directive 2005/36/EC On the Accreditation of the Minimal Requirements for the Preparation of Nurses and Midwifes and Mutual Recognition of Acquired Qualification. https://eur-lex.europa.eu/LexUriServ/LexUriServ.do?uri=OJ:L:2005:255:0022:0142:EN:PDF.

[6] Directive 2013/55/EU Of The European Parliament And Of The Council of 7 September 2005 on the recognition of professional qualifications.(2013). https://eurlex.europa.eu/LexUriServ/LexUriServ.do?uri=OJ:L:2013:354:0132:0170:en:PDF.

[7] WHO Regional Office for Europe (2000). Munich Declaration Nurses and Midwives: A Force for Health.

[8] The Act Of July 15th 2011 on the Profession of Nurses and Midwifes (Journal of Laws of 2016, item 1251 and 2020). http://prawo.sejm.gov.pl/isap.nsf/download.xsp/WDU20111741039/O/D20111039.pdf.

[9] The Act of October 30th 2017 on the Higher Education Law (Journal of Laws of 2017, item 2183). http://prawo.sejm.gov.pl/isap.nsf/download.xsp/WDU20180000345/O/D20180345.pdf.

[10] The Recommendation of the National Accreditation Council, Resolution No 103 / IV / 2017 of 22 June 2017. https://www.gov.pl/web/zdrowie/krajowa-rada-akredytacyjna-szkol-pielegniarek-i-poloznych-kraszpip.

[11] Sahu S., Lata I. (2010). Simulation in resuscitation teaching and training, an evidence based practice review. J. Emergencies Trauma Shock.

[12] Consell Català de Resusscitació (2010). Ressuscitació Cardiopulmonar. Manual de l’alumne.

[13] European Resuscitation Council 2015. https://cprguidelines.eu/guidelines-translations.

[14] Oteir A.O., Almhdawi K.A., Kanaan S.F., Alwidyan M.T., Williams B. (2019). Cardiopulmonary resuscitation level of knowledge among allied health university students in Jordan: A cross-sectional study. BMJ Open.

[15] Madden C. (2006). Undergraduate nursing students’ acquisition and retention of CPR knowledge and skills. Nurse Educ. Today.

[16] Kanstad B.K., Nilsen S.A., Fredriksen K. (2011). CPR knowledge and attitude to performing bystander CPR among secondary school students in Norway. Resuscitation.

[17] Özbilgin Ş., Akan M., Hancı V., Aygün C., Kuvaki B. (2015). Evaluation of Public Awareness, Knowledge and Attitudes about Cardiopulmonary Resuscitation: Report of İzmir. Turk. J. Anaesthesiol. Reanim..

[18] Hamilton R. (2005). Nurses’ knowledge and skill retention following cardiopulmonary resuscitation training: A review of the literature. J. Adv. Nurs..

